# Identifying the strengths, weaknesses, opportunities and threats (SWOT) of return-of-service bursary schemes for health workforce capacity: a qualitative study of Botswana, Lesotho and Eswatini

**DOI:** 10.1136/bmjph-2023-000142

**Published:** 2023-10-12

**Authors:** Sikhumbuzo A Mabunda, Andrea Durbach, Wezile W Chitha, Oduetse Moaletsane, Blake Angell, Rohina Joshi

**Affiliations:** 1 School of Population Health, University of New South Wales, Sydney, New South Wales, Australia; 2 George Institute for Global Health, University of New South Wales, Sydney, New South Wales, Australia; 3 Australian Human Rights Institute, University of New South Wales—Kensington Campus, Sydney, New South Wales, Australia; 4 Health Systems Enablement and Innovation Unit, University of the Witwatersrand, Johannesburg, Gauteng, South Africa; 5 Department of Public Health, Walter Sisulu University, Mthatha, South Africa; 6 Pharmacovigilance and Clinical Trials, Botswana Medicines Regulatory Authority, Gaborone, Botswana; 7 The George Institute for Global Health India, New Delhi, Delhi, India

**Keywords:** public health, education, economics, history

## Abstract

**Background:**

Investing in training citizens in return for service is a strategy used by Botswana, Eswatini and Lesotho to strengthen their health workforce. These strategies, known as return-of service (RoS) schemes, offer bursaries in exchange for future service. We aimed to ascertain the strengths, weaknesses, opportunities and threats (SWOT) of RoS schemes in these three Southern African countries to inform ongoing policy debates on the value of the schemes.

**Methods:**

Qualitative semistructured interviews were undertaken via Microsoft Teams to elicit the perspectives of policy-makers who administer RoS schemes in each of these countries. The interview guide was developed from a detailed literature review, and discussions with policy-makers and other researchers. Interviews were conducted over a 17-month period between November 2020 and April 2022. We used deductive and inductive approaches to thematic analysis. Furthermore, we conducted internal and external analysis of the emergent themes using SWOT framework.

**Results:**

We interviewed 9 policy-makers who had work experience that ranged from 5 to 22 years implementing the schemes. The organisational structure of the schemes was a strength compared with seventeen weaknesses, four opportunities and three threats. Prominent weaknesses are the outdated policy documents with some irrelevant and discriminatory conditions, rigid policies, failure to renew bilateral agreements, contextually different training from country of origin, high defaulter rates, poor coordination of schemes, poor monitoring and lack of evaluation of the schemes. Sustainability of the schemes in retaining health professionals is threatened by lack of funds. These schemes present opportunities to invest in effective information systems.

**Conclusions:**

While the intention of the RoS schemes were to educate the citizens, develop the economy through increased employability of the citizenry and build the health workforce, the schemes were poorly planned and coordinated and have never been evaluated. Weak information systems and failure to employ some RoS beneficiaries render the scheme unsustainable.

**WHAT IS ALREADY KNOWN ON THIS TOPIC**

**WHAT THIS STUDY ADDS**

- We find that the objectives of these schemes are often hindered by lack of evidence-based planning and implementation.
- Some beneficiaries are trained in contexts that are different from those in their home country, thus rendering them requiring significant retraining.
- Beneficiary compliance with contractual obligations are poorly monitored and the level of beneficiaries defaulting on their contracts is unknown due to poor coordination and poor information systems.

**HOW THIS STUDY MIGHT AFFECT RESEARCH, PRACTICE OR POLICY**

- We highlight key shortcomings of these policies that are limiting their impact and value to local health systems and identify key areas for potential intervention to overcome these.
- There is an urgent need to improve the policy planning and implementation processes including the development of more structured policies, with detailed evaluation plans and timelines for reviews, and improved information systems so countries are able to monitor beneficiaries across their training and service periods.
- This should be accompanied by strengthened global health workforce research to definitively determine the impact of these schemes in meeting their stated objectives.

## Introduction

 Low- and middle-income countries (LMICs) have the highest burden of disease and have the highest shortages of health professionals due to underproduction, maldistribution and emigration to higher income countries.^1^,^5^ In order to address this, the WHO developed the Health Workforce strategy 2030. The strategy emphasised the importance of taking account of labour market dynamics and education policies to address shortages and improve distribution of the health workforce to enable the best achievable improvements in health outcomes, social welfare, employment creation and economic growth.^6^

Under interim targets for 2020, member states were to develop local coordination mechanisms to implement the human resources for health (HRH) agenda and introduce registries to ensure visibility of the HRH stock, their education, distribution, movements, capacity, demand and remuneration.^6^ The HRH global strategy is aligned with the United Nation’s Sustainable Development Goal (SDG) 3, which advocates for a substantial increase to health financing and the recruitment, development and training and retention of the health workforce (target 3c) especially in LMICs.^6^,^8^

Governments need reliable support and partnerships from the international community and the tertiary education sector to ensure that there is adequate funding and available training platforms for this needed increase in production of the health workforce.^9^ As the world gears up for the SDG interactive Dialogue and Summit in March and September 2023, respectively, it is important to review some of the health workforce strategies used by some LMICs. Botswana, Eswatini and Lesotho are three Southern, sub-Saharan African countries that experience a high burden of maternal and child mortality and communicable diseases, and an increasing non-communicable disease burden.^9^,^14^ These three countries each have a physician to population ratio of below 0.5 per 1000 population and a midwife to population ratio of below 4.2 per 1000 population.^4 6 15^ The combined ratios of doctors, nurses and midwives for these countries is well below the 4.45 per 1000 population target specified under SDG 3c for the attainment of universal health coverage.^4 6 15^ As of May 2023, Botswana, Lesotho and Eswatini showed stagnation with persistent challenges on attainment of the SDG 3c targets.^16^

The governments of Botswana, Eswatini and Lesotho Invest substantial resources in training their health workforce and prioritise health workforce training.^23 9 14 17^,^32^ Educational investments predominantly made throughreturn-of-service (RoS) schemes are health workforce planning strategies that contract a beneficiary to stipulated number of years of government service.^2^,^4^

All three nations largely rely on neighbouring South Africa and other countries for specialist medical and specialist nurse training.^33^ While Botswana has had a medical school training medical students since 2009 and pharmacists since 2018, internal capacity constraints means that the country has had to send pharmacy and medical students to other countries for training.^30 34^ Both Eswatini and Lesotho rely on other countries for most health sciences programmes except for pharmacy (in Lesotho) and nursing.^23 9 26^,^28 30 34^

RoS schemes are used widely across the world in countries of all income levels,^435^,^44^ though the exact number operating such programmes are unknown. There is limited published evidence of the operation or impact of these and that which exist is predominantly focused on high-income nations, though some limited literature has emerged from schemes in South Africa, Sri Lanka, Philippines, Malawi and Zambia.^435^,^44^ Until 2023, there was no literature on the three countries’ RoS schemes.^33^ The RoS schemes in Botswana, Lesotho and Eswatini have not been evaluated and there is very little literature about their effectiveness in these countries, reflecting a similar lack of literature globally.^33^ There is no literature from these three countries quantifying beneficiaries who were funded, those who fulfilled or served part of their contractual obligations, or those who defaulted their contracts. This has left a gap in understanding the operations and value-add of RoS schemes and makes it difficult for policy-makers to learn from each other’s successes and/or failures for improved implementation. Assessing the internal and external context of RoS will enable a greater understanding of the strengths, weaknesses, opportunities and threats (SWOT) of these schemes. This is vital to ensure that the schemes are meeting their aims, inform policy developments and improvements and consider their relative performance against alternative training schemes to build health workforce capacity. It will also help ascertain if the countries are on track to attaining SDG goal 3 (target 3c), aligned with the WHO HRH 2030 strategy or if they need to review their strategies for a sustainable health workforce solution. This study therefore aimed to close this literature gap.

### Context

The three countries have three distinct systems of governance with Botswana being a Democracy (President head of country), Lesotho a Parliamentary-Constitutional Monarchy (Prime Minister as head of country and King as ceremonial head of country), and Eswatini an absolute Monarchy (King as head of country).^45^,^47^ The schemes are divided into preservice and in-service programmes depending on whether individuals are school leavers or employed in government.^33^ In-service schemes are strictly reserved for all government employees in Lesotho and Eswatini, and for the specific employing ministry in Botswana, for example, the Ministry of Health.^33^ Preservice schemes are reserved for non-government employees, mostly youth who have just completed high school.^33^ In Botswana, preservice RoS bursaries are administered by the Ministry of Education and in-service schemes are administered by the Ministry of Health’s Human Resource Development department. In Lesotho, all schemes are administered by the National Manpower Development Secretariat (NMDS). In Eswatini, preservice schemes are administered by the Ministry of Labour while in-service schemes are administered by the Ministry of Public Service.

## Methods

### Design

Qualitative semistructured interviews were undertaken to understand the structure of RoS schemes and how they are implemented to address the shortages and maldistribution of the health workforce. Participants were interviewed as individuals or as a group depending on their preference. This article is reported inline with the Consolidated criteria for Reporting Qualitative research (online supplemental appendix 1).^48^

### Sample and recruitment

Senior bureaucrats (policy-makers) responsible for the implementation of six bursary/scholarship schemes (two from each country) that train health workers in RoS were approached for interviews using purposive sampling. Accounting officers were approached to request for the identification of participants. Participants were included if they were managing any RoS processes (design, planning, budgeting, implementation and/or monitoring) government RoS schemes for at least 2 years in the three countries. All invited participants accepted the invitations. Six interviews of nine participants were held between the 17 November 2020 and the 07 April 2022 based on their availability. The nine participants were representative of the most relevant individuals who managed RoS schemes in the three countries. Once we had identified this population, no further assessment of thematic saturation was conducted.

### Data collection and data analysis

This section is described in online supplemental appendix 3.

### Reflexivity, patient and public involvement, and ethics approvals

This section is described in online supplemental appendix 3.

## Results

There were four respondents from Botswana, three from Lesotho and two from Eswatini. Of these, five were male. Table 1 summarises the Strengths, Weaknesses, Threats and Opportunities of these schemes.

**Table 1:** Summary of return-of-service schemes of Botswana, Eswatini and Lesotho using the SWOT approach

	Internal		
Positive	Strengths Structure of RoS schemes Configuration of schemes Centralised governance Continuity and policy certainty Low turnover of custodians Training of health workers is deemed to be critical to improve access to healthcare Some beneficiaries might not have had the opportunity to afford tertiary education Botswana and Eswatini have special provisions for individuals from lower socioeconomic groups and those with a disability	Weaknesses Available budget rather than population needs is the key driver of bursary decisions Outdated policy documents Rigidity of policies Failure to renew agreements Shortages of staff to manage RoS schemes and health programmes Contextually different training from country of origin/practice and adaptive health system Weak National Registration systems Lack of interoperable information systems Limited mechanisms to prevent defaulting of schemes Failure to retain beneficiaries Training professionals for other countries Ineffective sanctions for those who breach their contracts Poor governance and coordination between implementing departments and units Schemes do not address equity Lack of evaluation of programmes Paradoxical shortage of skilled health professionals and failure to employ beneficiaries Discriminatory practices	Negative
	Opportunities Having a trained workforce Commodifying the health workforce for financial remittances with training or high-income countries. Invest in information systems Opportunities for engagement	Threats Dual structure of the health system—working in the private sector Migrating to a high-income country or other country Programme goals hindered by lack of funds	
	External		

SWOTstrengths, weaknesses, opportunities and threats

### Strengths of RoS schemes in Botswana, Lesotho and Eswatini

A number of key strengths of RoS schemes were identified by respondents. They recognised that long-term workforce capacity needs to be developed through a combination of RoS schemes for government employees (in-service) and those for aspiring health employees (preservice). Running these schemes at the national level allowed national governments to clearly articulate national targets/plans. The programmes also foster cooperation between government departments since the programmes are planned for all sectors of government simultaneously. The policies have been in existence for more than 20 years, making it easier to administer. Respondents have been responsible for the administration of these schemes for between 5 and 22 (5–22) years making it easier to preserve institutional memory.

#### Structure of ROS schemes

The three countries have two broad RoS programmes that benefit the Ministry of health, namely, preservice programme for aspiring health professionals who have completed high school and the in-service programme for public sector employees who are upgrading their skills (undergraduate or postgraduate). The preservice programme is coordinated by the Ministry of Education in Botswana, Ministry of Labour in Eswatini and the NMDS in Lesotho, who are also responsible for the in-service programme. In Botswana, the in-service programme is coordinated by the Human Resources Development unit of the Ministry of Health and by the Ministry of Public Service in Eswatini.

Beneficiaries are contracted through a process of bonding, to serve the country’s health system on completion of their studies. Programmes fund all costs related to studies including transportation from the capital city, tuition, accommodation, stationery and sundry. Beneficiaries serve a year-for-year service contract based on the duration of funding support received if they were preservice beneficiaries in Botswana and Eswatini. Both sets of beneficiaries in Lesotho have to serve for a minimum of 3 years if funded for a year, and for twice the number of years of funding support if funded for more than a year. In-service beneficiaries in Botswana add a year of service to a period of funding support that is between a year and 5 years, with the maximum service period capped at 5 years.

In general, in-service beneficiaries in both Botswana and Eswatini, and Botswana’s preservice health sciences beneficiaries do not need to repay the monies spent on them for their studies after completion of their studies unless they default (that is, they do not serve their full required period after studies). However, non-health sciences preservice beneficiaries in Botswana who are not on the top achievers’ programme have to repay 100% of the funding support. Preservice beneficiaries in Eswatini repay 50% of the grant within twice the number of years of funding support. All Lesotho’s beneficiaries repay 100%, 65% or 50% of the grant depending on whether they, respectively, do not return to the country at all, return to the country but work in the private sector or return to the country and work in the public sector. In Lesotho, repayment has to be within 5 years of completion. These repayments are in addition to the service obligation.

During the period of absence, in-service beneficiaries are expected to return to their posts after completion of their studies. While implied and preferred, the contracts for preservice beneficiaries do not necessarily have to be fulfilled in the public health sector as long as the beneficiary works within their country’s borders.

**PP2:** …the scholarship…, it’s like highly competitive, 50% of it … is grant, and 50% of it is… a loan. So, like, once, you get the scholarship …you start studying. So, government is saying, you know it’s for example…, for the entire period you are going to need, say, 200,000…, You only pay back hundred, the other 100 000. If you spent four years, then the pay-back time is twice that period. If you spent seven years, it’s twice that period because …, that’s how it was structured.

**PP1:** So, if an officer for example goes for a two year Master’s degree. The bonding means after you have completed your studies and you are reinstated back to work…, you need to serve at least three years…, in a government setting.

**DS3:** …there is a certain duration that you are bonded to because of the period of your study, it differs. … if you are going to school for five years…, you are going to come back and serve for 10 years before we can release you.

Beneficiaries are either trained locally, in fellow African countries (with South Africa being the most popular), or abroad (eg, Australia, Canada, UK, Taiwan, Russia, Ukraine, etc).

### Weaknesses to policy implementation

There are several challenges in the implementation of the RoS schemes. These include, non-completion of studies, weak administration, poor coordination, contract default, including not returning to the country, and poor retention of beneficiaries.

#### Available budget rather than population needs is the key driver of bursary decisions

The number of beneficiaries that can be funded in a year depends on the allocated budget, and the available spaces for study, and not on the health workforce needs of the country. Availability of admission is often a product of bilateral negotiations and agreements with governments and/or universities of other countries. The policy will not consider the country’s needs, that is, shortages of health specialists, need for public health trained specialists, need for specialists trained in management practices (eg, with a Master of Business Administration), etc.

**DS3:** So, my question is, so, what happens now to the current need that we have because we don't have specialties in our country… so, the gap that I see, or the advice that I would give them is to actually look into medicine differently. I don't know whether other disciplines or other, uh, fields share the same sentiments but for us because there is a gap in our country, you need to allow doctors to specialise in both. You cannot say you cannot, uh, have somebody do another Master, because even some Masters are related.

The RoS custodians allocate funding, and the Ministry of Health is then requested to work within the allocated budget to fund priority academic programme beneficiaries. These priority areas are determined based on operational service needs and not evidence based (without research inputs). As a respondent states:

**DS3:**…within that limit, they do not come to us and prescribe how many we can have, but they will say: ‘Ministry of Health I only have this amount of money… so, what are your priorities?’. And then we would stipulate our priorities. We would say: ‘ok, this is the ideal thing that we want… and it has this cost, but because we have this much now then we will maybe only continue with people that are enrolled or if it allows new people… then we would say within the one that allows us to recruit new people, our priorities will be doctors, nurses and what.’ So, we choose from there and their responsibility is now to create the position.

#### Outdated policy documents

Policy documents have not kept up with changing human resource (HR) practices. The policy documents are from the years 1977 for Eswatini’s preservice beneficiaries, 2000 for Eswatini’s in-service beneficiaries, 1995 and 1996 for Botswana’s preservice and in-service beneficiaries, respectively, and 1978 for all Lesotho’s programmes. None of these policies have been reviewed or evaluated which presents a challenge as other dependent HR practices have evolved as narrated by a participant:

**GBL1**: The same thing with the bond… they go hand in hand in terms of age. So, what is happening over time, some HR [Human Resource] principles, they changed (sic). Like, I can give an example. At one point the government took a decision to say when you are on studies, before, the practice, this is how it was done. I would go for one year, let’s say…, I was approved for a four year programme and… the first year I would earn my full salary. The second year up to the 4th year, I will be earning half salary. But, of recent, I think around 2006/2007…, government took a decision to say: ‘no, we need to align, uh, with international HR practices because you didn't go there voluntarily’. You went under government obligation, which means why should you be denied your full renumeration (sic)? So, Government decided: ‘ok, fine, uh, anybody who goes on government sponsorship, they will be given full salary irrespective of the number of years that you go for’ (sic).

The latter example was an operational change that was not documented in the policy documents.

#### Rigidity of policies

Generic policies that cater for diverse Ministries’ needs have inherent rigidities which impact on efficiencies of the programmes. An example is Lesotho’s policy which sponsors programmes in a hierarchical manner from a Diploma, Bachelor, Honours, Masters to a Doctoral qualification. A challenge is when a clinician (dental or medical professional), for instance, decides to undertake a Masters qualification at some stage after their Bachelor qualification. As a clinician, this means that they can no longer be funded for a clinical specialty (as it is also a Masters) under the government programme but can only then be funded for a Doctoral qualification.

#### Failure to renew agreements

Lesotho had agreements with South African universities on a quota of students they would send to South Africa annually, but such agreements have not been renewed for more than 15 years.

**DS1:** …and on our side I think it’s just uh, an issue of not … renewing agreements because the agreement was made sometime in 2005, 2006 and it hasn't been revisited. So, I guess as institutions staff is changing… and some now are not aware of the agreement, so the only universities who are still with us on that are UOFS [University of Free State] and Walter [Walter Sisulu University].

#### Shortages of staff to manage RoS schemes and health programmes

Administration of the schemes also requires adequate staffing in terms of numbers and skills. This is unfortunately often lacking. The realities on the ground are such that the most experienced clinicians have to juggle between clinical work and administration sometimes with little training on administration. Removing them from clinical areas also means a gap left behind in the clinical area. Because of being senior, they are also expected to contribute the most to health planning and strategy setting which also need an academic foundation and support.

**DS3:**…‘I was a, I was a, uh, an orthopaedic surgeon but now I'm frequently called in the ministry to do epidemiology, issue statistics and epi [epidemiology] and financial related things to advise on that’. So now, I end up saying no. It looks like I need to have a skill in MBA [Master of Business Administration] or Public Health.’

#### Contextually different training from country of origin/practice and adaptive health system

One respondent believed that training should be universal for a similar programme regardless of the country of training. For instance, a medical curriculum in Botswana should be similar to that of the UK, Australia, Taiwan and everywhere else in the world. However, training of beneficiaries outside their own countries sometimes results in differences between the training and the practice environment in their countries of origin. This at times results in beneficiaries who are unable to complete their duties or serve in their local context as was experienced by Eswatini.

**PP2:** Let me make for an example, here we have had students that have trained in Taiwan…, you know, and some students that are trained in Ukraine. Uh, some of them it has been difficult to re-employ them in the sector (sic)…, because you know the equipment that is used here and the processes that they use here, is somehow different from… their training? …they had spent the required number of hours in… a hospital kind of environment… but now the training, compared to the actual realities on the ground are two worlds apart. So, it was then difficult to absorb those kind of students… into employment.

It is also important to note that health professionals’ curricula and training methods need to evolve and adapt to the country’s health system. An example was given by one respondent:

**PP2:** …in one of our studies, you know… we realised that we were training, uh, so many health professionals in the curative point of the spectrum. So, we wanted to focus more on the…preventive…you know aspect of the spectrum… particularly…, in the nursing profession. To say, you know, could we invest more in Community health training…, and maybe, yeah, less on curative nursing… we are still like fighting with the Ministry of Health and we have been having the discussion for four years now.

#### Weak national registration systems

Lesotho has no street addresses, and this makes it difficult for locating individuals post completion. Until 2014, Lesotho only used passport numbers as unique identifiers of citizens. The challenge was that passport numbers changed at the expiration of the passport. Surnames of female beneficiaries often changed after marriage, and not all married individuals had marriage certificates, and that also made it difficult to know the individuals who benefited from government schemes.

**DS1:** People only had passports and very few of them had passports. We only started issuing out IDs [identity documents] in 2014. So, right now we are working with the Ministry of Home Affairs and that will also assist us in tracing wherever you are. Your ID… will still be the same. …but up to now, we cannot say these are the steps that we have taken because we don't have data. Even what we have here, you will find that: ‘ok, my name is DS1, I get married and once the person gets married you have lost them. But now that everything is, the Ministry of Home Affairs is linking all the information, then we will be able to interface with that and then it’s only then that we will be able to trace. Previously we couldn't.

#### Lack of interoperable information system

None of the countries has an information system that links beneficiaries allocated for study opportunities, the tertiary institution where they are/were, the finances disbursed for that beneficiary’s studies and the allocated service area. One respondent referred to this phenomenon of weak information systems as information asymmetry because the left hand does not know what the right hand has.

**PP2:** …but in terms of really monitoring them in a systematic way ……we don't have a system to actually track all our trainees to say these ones, like trained in health…, where are they? What are they doing? Are they coming back to the system or are they not? We don't have that kind of a system. I wish we did have one because it will be helping us quite a lot. So, now we just depend on them… to say: ‘Yes we are back’…

#### Limited mechanisms to prevent defaulting of schemes

Defaulting of schemes is a common occurrence and is at times related to the high unemployment rates and low government wages. In the cases where there are repayment obligations, unemployed beneficiaries also cannot fulfil this obligation. At times, there are unconventional methods used to define defaulters such as is the case in Botswana’s preservice programme.

**FZ1:** Remember, the ones who default in our, the way we define it, our defaulters are those who started paying but then stopped.

This means that those who complete their studies, leave the country or stay on in their host countries where they studied would not be considered to be defaulters.

**FZ1:** I think…, basically because the reason why in any case, most students tend to stay abroad is because one, there are no jobs, or the jobs are not paying enough. I think the government, we need to look ourselves internally on this.

Eswatini’s Ministry of Labour estimated the defaulter rate to be over 50%. Some beneficiaries feel entitled to receive the funding support for their studies and therefore do not feel the need to fulfil their contractual obligations.

**DS1:** It’s a very huge number, you know, like now, everyone will be telling you that there is issues of employment (sic). Like we are, it’s obvious to everyone that there is issues of employment (sic). But even to those who are employed, it’s still not, it’s not a priority for them… a person would rather buy a car than repay the NMDS [National Manpower Development Secretariat] money. So…, It’s a large number.

**PP2:**Yeah…, in the recent years, you know the defaulting probably is due to a number of factors one of them is unemployment…

##### Failure to retain beneficiaries

Several factors have been found to be responsible for failure to retain beneficiaries ranging from incentives, working environment and lack of administrative support. Some beneficiaries, especially those trained to specialise get frustrated by the system due to lack of equipment, poor salaries and lack of support and end up resigning before fulfilling their contractual obligations. Professionals often leave prematurely due to poor working conditions and lower salaries and benefits.

**GBL2**: Yeah…, talking about retention because those are the two other things that really affect…, the salaries, the equipment and then also generally to be able to cater for all that is needed when they complete the programmes. …most of them or half of them, are ready to serve back, but once they have these things…, not being arranged…, instead of serving them, now they get these frustrations.

Some people do not like working in rural areas. A beneficiary with a very good skill can decide to quit government just because they do not like or want to work in rural areas.

##### Training professionals for other countries

The poor incentives, working conditions, poor planning and poor monitoring of the schemes also lead to training of health professionals for other countries. Despite Lesotho’s initiative to train dental therapists (mid-level dentists) from 2016, they have not been able to recruit any of the three cohorts and some have even been released to seek employment in South Africa.

**DS3:** So, we started training these people in 2016 and then I think we have like three cohorts now that have come out…, but we have not been able to recruit them into our system because us as government we are not able to help them, and our private sector is also not huge to accommodate them… they ended up asking for letters to be released into South Africa.

Such examples extend to the other countries as well:

**GBL1**: …that is a fact. We are not able to retain our medical specialists. We are not able to not necessarily retain only the medical specialists, even the very nurses who have gone to specialise as neonatals (sic), who are specialising as, uh, emergency or trauma nurses…, are leaving for the private sector, they are leaving for the parastatals because they pay much better. Their salaries are doubled. Some are even leaving to South Africa for better pay structures. They are leaving to Namibia, they are leaving to even the US or the first world countries.

Eswatini’s Ministry of Labour has an optimistic outlook at this occurrence. They believe that the beneficiary who chooses not to return could learn more valuable skills for their home country which could later be transferred to other locals in their home country. They also believe that this could present an opportunity for the two governments to negotiate remittances.

**PP2:** … if the graduate, you know, decides to stay and work in South Africa, I mean, we are not so much fussy with that because we still believe we are going, somehow going to get something back from that graduate in terms of remittances in terms of all other things you know…,

##### Ineffective sanctions for those who breach their contracts

Defaulters are required to repay government for all expenses. Lesotho is in the process of introducing interests to the repayment amount.

**DS1:** So, now we have introduced things like interest and penalties for defaultees (sic). Now, we don't have any type of penalties for defaultees (sic). So, you can default and then come back to us when you want to go back to school, we accept you back. As long as you end up paying.

### Poor governance and coordination between implementing departments and units

These schemes rely on the strength and objectivity of the existing leadership at all levels of government and governance. Some of the processes are subjective, as a result some ideas might gain favour with one set of leadership and not gain favour with another.

**DS3:** …it depends on who has you know, sometimes, uh, our, let me say our African leadership… it suffers a lot from sometimes, uh, individualism whereby somebody would look into whether they are interested or maybe they affiliate such issues to politics. Now, it depends on somebody else whether they like you or not. …you really have to have an objective leader for you to… get such maybe, uh, endorsement.

There is poor coordination between the funding department or sphere of government and the Ministry of Health (which employs beneficiaries). At completion, beneficiaries are to introduce themselves to the potential employers and state the qualifications acquired. Their employment depends on the presence of a vacancy. Even though Botswana’s in-service programme is coordinated from within the Ministry of Health, it also has challenges coordinating with their employing counterparts.

**GBL2**: Well, I think… there is no much relation (sic)… even though we are one body. The reason why I'm saying that is that, uh, the very example that I have just given you…, there is no clear relation… within the four years that we are training GBL3…, we need to prepare for him, such that when he completes, he goes straight into the position and serves the government of Botswana.

**PP2:**Yeah, the Ministry of Health…, it’s quite difficult man to deal with the Ministry of Health. Difficult in the sense that I sometimes feel they are…, not helpful enough in some instances, getting things done…

**DS3:**… but there isn't any professional or a standing meeting… that is routine in terms of informing, uh, Manpower [National Manpower Development Secretariat]…

### Schemes do not address equity

Despite the recognition that children from wealthier families are likely to attend well-resourced schools and therefore obtain higher grades that will stand them a higher chance of being granted government sponsorship, the three preservice programmes assess applicants from low socioeconomic groups the same way as those from wealthier families. Botswana’s wealthy students studying in local private schools or other countries will for instance be assessed the same way as rural origin students from low socioeconomic backgrounds. However, Orphans and Vulnerable children (includes those with a disability) have a special dispensation under the office of the president. These beneficiaries are assessed by social workers who compile a means assessment report. These beneficiaries are for instance assessed at five points lower (31 vs 36) than other applicants. Beneficiaries from underprivileged backgrounds in Eswatini must either be those who have both their parents deceased or those whose parents relied on some or other social support for the potential beneficiary’s education throughout primary and/or high school. Lesotho is now considering the inclusion of affordability scores in its assessment of candidates to ensure that the sponsorship is limited to those who would not otherwise have been able to afford university fees.

**DS1:** There are students who pass better because they are from more resourceful schools. Yes, there are students who have potential but could not perform to their maximum because they are from a rural school or they are from a less resourced school. So, now we are looking at changing our target. We are saying we are not only sponsoring you because you have passed, we also want to sponsor according to affordability. We don't look at whether your parent is a billionaire or not. If you have been submitted for sponsorship, we sponsor you.

### Lack of evaluation of programmes

None of the programmes have been evaluated for impact despite the desire to do so in the past. Respondents have opinions that the programmes seem to have had an impact but there have been no formal processes to confirm that.

**DS1:** …we cannot say it was actually evaluation, it was, yeah…, people’s opinions on the policy, … but it hasn’t been…, there is no document that you will find that says this has been evaluated, but steps have been taken previously to evaluate and nothing has gone through.

**PP2:** No, we have never evaluated the programmes. You know, honestly, we have not, you know. But I think we are making an impact.

### Paradoxical shortage of skilled health professionals and failure to employ beneficiaries

Despite the known shortages of skilled health professionals in health facilities in Eswatini and the presence of vacant but unfunded posts in some health facilities, there are still skilled health professional who cannot be employed. Eswatini conducted an unemployment survey in 2016 and found that up to 7.5% of their beneficiaries were unemployed. Those unemployed includes pharmacists and medical doctors who were trained outside the country, and who are considered to be scarce and critical skills because there is not enough money to pay them. There is hope that if all funded beneficiaries were placed in employment, then defaulting could be minimised.

**FZ1:** So, people sometimes don't default because they really want to default, it is because there is nothing in the open for them. So, if we are able to place every student and find employment for them, maybe then maybe… [they should be released from their bods].

### 
Discriminatory practices

Some practices are discriminatory against women, for instance the pausing of sponsorship of all pregnant women beneficiaries under Botswana’s grant-loan scheme from the fourth month of pregnancy (regardless of the expectant mother’s health state or preference) until after birth of the child. Similarly, female beneficiaries who are found to be pregnant before embarking on international studies are forced to not travel for studies until after birth of the child.

### 


Threats to the effectiveness, sustainability and impact of the programmes

#### Dual structure of the health system—working in the private sector

Due to having a dual health system, some beneficiaries who move to the private health sector after resigning from government health services feel that they should not repay the government bond since they are still serving their country’s population as cited by participants in Botswana. This matter partly arises due to flexibility of the contract which does not directly prevent beneficiaries from serving the private sector. While the country retains the health provider, working in the private sector impacts access to those who cannot afford services in the private sector.

#### Programme goals hindered by lack of funds

The programmes’ aspirations were sometimes over ambitious to the extent that the public thought the programmes were ineffective as they did not always train, reskill and upskill professionals at the expected and/or promised rate. This is due to lack of resources and/or diminishing budgets seen over the years.

**FZ1:** More than anything is the budget, because, with the economy now, with our country not making as much money as we used to, now we are having budget…constraints, and also, we want to expand and sponsor more students than we currently are sponsoring right now. So, we are reviewing this policy based on that.

**DS3:** …I think in the last two, three years or so there has been a big challenge regarding Manpower [National Manpower Development Secretariat] sponsoring people who are now going… to study because some people have disappeared and never really gave back to the government.

#### Migrating to a high-income country or other country

Following their training, some beneficiaries work for the public health sector for some time within their countries and later migrate to either their training country or to a higher income country than their own country.

### Opportunities to leverage from these schemes

#### Having a trained workforce

These RoS schemes creates an opportunity for the development of a trained health workforce, at times even when the funding is not directly from government. For instance, Lesotho’s policy is clear that all funding received by their nationals would be considered to be from government irrespective of the source (eg, funding from individually sourced scholarships outside Lesotho).

#### Commodifying the health workforce

Eswatini’s Ministry of Labour would like to explore opportunities of how the schemes can integrate with other global economies in terms of them training and sending their skills in exchange for intergovernmental remittances. This would require them to increase production of the health workforce for export.

**PP2:** I think we need to actually even look at… how we can integrate with other economies of the world in terms of, you know, training and sending our skills…, with broader skills requirements not only in… Southern Africa, but globally… Because for me, I'm saying you know, like if we are going to talk about globalisation, maybe let’s not talk about globalisation as something that will benefit…uh, the western industrialised countries only, but let it be Globalisation that benefits you know, uh, the South as well. In terms of, our competitive advantage as the South is its skills. So, I think if we can honestly focus on perfecting our skills development programmes… and then exporting these skills to other countries, yeah, we can get quite a substantial returns (sic) on that… in remittances and all other things, and cultural sharing experiences, and so on and so forth.

#### Invest in information systems

The presented information systems challenges presence an opportunity to invest in and innovate interoperable information systems that will correct the asymmetries.

#### Opportunities for engagement

Having the programmes coordinated between sectors encourages engagements between Ministries and enables the understanding of inter-Ministerial strengths and capacities to avoid having to seek consultancy assistance in instances where other Ministries could assist.

## Discussion

In this study, we found that RoS schemes are national government priorities whose policies have been in existence for at least 22 years without being reviewed. The aim of RoS schemes is to strengthen the health workforce while providing opportunities for the populace in higher education relevant for the needs of the country. If implemented well, the RoS schemes could have a significant impact in improving health outcomes and address social inequity in the country. However, the lack of good governance, adapting the policy to the current context and poor implementation has led to weakness of the RoS schemes, likely limiting their impact in practice. In all three countries, we found that the policies have failed to keep up with the current population and beneficiary needs, they are poorly coordinated, lack information systems, are poorly monitored and have not been evaluated. The RoS schemes are based on the available budget rather than evidence and it is unclear that they represent the most effective use of scarce available resources for health. These schemes are further weakened by failure to employ and retain beneficiaries. The countries have high defaulter rates (estimated to be over 50% at times), diminishing budgets, contextually unprepared beneficiaries. Overall, the weaknesses and threats of these beneficiaries seem to outweigh the strengths even though it is hard to definitively say without a formal, robust evaluation.

There are flaws throughout the value chain of the implementations. Due to poor planning, some funded beneficiaries are not employed due to lack of funds and thus make it seem as if health systems have excess HR for health while in reality, there is an acute shortage.^7 9 10 14 17 27 28 30 34 49 50^ Exploring options of commodifying the health workforce might therefore be premature until there is evidence that the population has adequate access to health workforce. Even then, the health workforce that can be exported should be limited to those who have been proven to be in excess, not merely a blanket agreement for the export of any cadre of health workers that are required by other countries. Models of commodifying health workforce are present in a number of contexts including the Philippines, Jamaica, Cuba and India.^51^,^61^ However, these remittances do nt always translate to benefits for the health system, but are sometimes channelled or redistributed to other national programmes.^62^

The weaknesses identified are not necessarily failures of the policy (RoS schemes) intentions but rather failures to plan and implement the schemes optimally. Some beneficiaries therefore exploit these loopholes to render the schemes ineffective, hence the high reported defaulter rates. These results suggest that the policy intentions could be to increase the pool of educated and employable individuals than to increase public sector capacity, for example, public health sector. Retention into the public health sector then becomes an added bonus for governments.^918^,^22 24 25 27^ This is because beneficiaries are responsible for finding their own jobs, and even then, employment is not guaranteed. This therefore relies on the honesty of beneficiaries whose outlook on life could well be different after completion of their studies as previously reported in a similar South African study.^4^ Even though outdated, the planning processes for RoS schemes do not incorporate the HRH strategies for their countries when planning for the health sector.^26^,^2834^ Furthermore, even though the perceptions of policy-makers and beneficiaries on the implementation challenges and success of Eswatini’s in-service programme were previously documented for three Ministries including Health by a Master of Social Science student (Public Policy), the participant from the Ministry of Public Service did not refer the researchers to the resource^25^ suggesting that the participant either did not know of it or did not think it would be important to share with the researchers. The researchers only found it after the interview.

This study further reaffirmed the established fact that health workforce interventions work best when there is concurrent health system effectiveness and universal health coverage.^1^ A Taiwanese interrupted time-series which examined the distribution of the health workforce 24 years before the implementation of National Health Insurance (NHI) and 8 years after the implementation of NHI (1971–2002) found the distribution of health providers to be improved significantly by the intervention.^1 63^ This therefore suggests that health workforce interventions like RoS schemes can only be as effective as the health system and cannot be viewed in isolation from other strategies (ie, targeted recruitment of beneficiaries (eg, a quota for rural origin beneficiaries), rural allowance, community service, spousal benefits, etc).^1 3 49 64^ Even though these three countries have three distinct government structures, their health systems and structures of their RoS schemes were similar.^916 45^,^47 50 65^

Poor working conditions, unattractive work environments and poor incentives are some of the reasons cited for beneficiaries to default their contracts, a significant weakness currently hindering the effectiveness of the RoS schemes. A solution to these challenges requires multi-Ministerial and multisector (public and private, etc) consultations and collaborations.^1 3 49 64^ This aligns with the milestone for an intersectoral HRH agenda in the WHO HRH workforce 2030 strategy.^6^

Working conditions could for instance be improved by having adequate supervision, developmental support, adequate staffing to reduce burnout, supply with adequate instruments and consumables, and referral support. Working environments can be improved by collaborating with other sectors to improve access to good education facilities for children, reduce crime, improve road infrastructure, improve prospects for job opportunities for spouses who work outside the health sector, avail entertainment facilities for families, and so on.^1 3 49 64^ Furthermore, recruiting beneficiaries from rural areas and the presence of rural elective placements could increase the likelihood of retention.^1 3 49 64^ If funds are limited, it might even be better to prioritise some of these issues rather than more RoS beneficiaries as the above issues will lead to the retention of an already trained health workforce (whether RoS beneficiaries or not).

Incentives could include free or subsidised accommodation, increasing incentives for working in unattractive and/or underserved areas such as the Zambian Health Worker Retention Scheme, South Africa’s rural allowance programme and Sri-Lanka’s priority school admissions incentive for beneficiaries.^1 38 43 64 66 67^ Governments should also review the salaries paid to health professionals in relation to the economy and other countries. It is also important for governments to be bold and consider other financial incentives that might not have as yet been implemented, for example, a once off bonus at the completion of a certain period of preagreed service in a specified health facility.

Though not perfect, Sri Lanka’s priority school admission programme for children of RoS beneficiaries has had a retention rate of 87% and could therefore offer valuable lessons to other LMICs like these countries under study.^43^ Likewise, a clear description of the anticipated service area could help improve the return on investments of RoS beneficiaries as is the case in Australia, Canada and India.^468^,^72^

**Figure 1:**
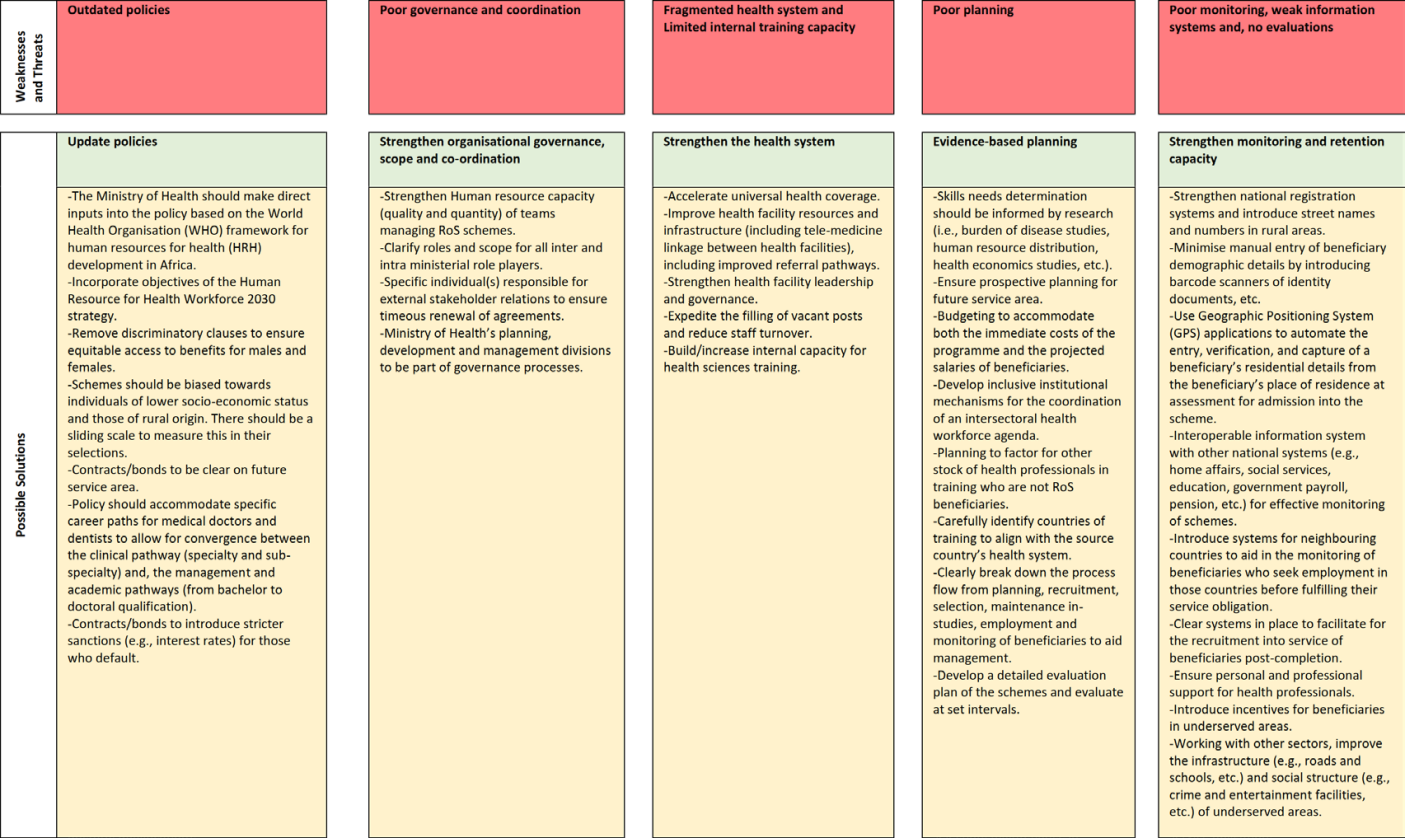
Recommendations for successful return-of service (RoS) implementation based on the strengths, weaknesses, opportunities and threats analysis.

Health workforce needs to not only be quantitatively adequate, but they should also be fit for purpose. It is therefore important for governments to ensure that countries where beneficiaries are trained have similar health systems to minimise costs of training the cadres. As demonstrated by Eswatini’s cadres where health professionals were trained in Ukraine and Taiwan but were found to be contextually incompetent. In recognising the differences in the health systems, South Africa’s Cuban trained medical doctors are reintegrated in a South African medical school for a further 12–18 months before being allowed to graduate and practice.^73 74^ This system is, however, expensive as the duration of training is increased and there is no guarantee that the medical student will pass in South Africa.^73 74^ It is therefore desirable that as countries build international relations, they also build their own internal capacity to train the health workforce in a familiar health system. Figure 1 summarises key recommendations that emanated from the SWOT analysis.

This study had some limitations. We interviewed nine stakeholders from the three countries and did not interview all stakeholders in the value chain. The stakeholders who were interviewed were very experienced, more knowledgeable and more informative. Even though we missed some important stakeholders like the Ministry of Health in Eswatini and, the Ministry of Health Human Resource Management in Botswana and Lesotho, within the constraints of a qualitative research study, this study can only present the view points of the research participants and recognise that other individuals may have different views. We did not test for thematic saturation; however, given we were able to recruit our target population we do not think this would have had a major bearing on the results. The SWOT framework is a subjective framework and a fairly simple tool which does not consider the broader sociopolitical context.

## Conclusions

RoS schemes are government strategies aiming to develop an educated citizenry, improve employability of citizens, develop the economy and build internal HR capacity for existing government employees. RoS beneficiaries in the health sector should therefore improve access to healthcare and result in an improvement of health outcomes. While the RoS policies may be well intentioned, stakeholders involved in these policies suggest that they are inadequately governed, planned and implemented leading to poor retention of the beneficiaries. Participants in this study further suggested that these planning and implementation shortcomings have worsened access to health professionals. Building health workforce capacity to meet objectives of the HRH strategy for 2030, and the SDG 3 targets requires improvement and integration of health workforce planning. These strategies require a whole of government approach, a coordinated approach and should be regularly evaluated. Furthermore, these programmes can only be effectively implemented if the information systems are strengthened to minimise waste. Such information systems need to be interoperable with other government information systems, with minimal manual entry.

## supplementary material

10.1136/bmjph-2023-000142 online supplemental file 1

10.1136/bmjph-2023-000142 online supplemental file 2

10.1136/bmjph-2023-000142 online supplemental file 3

## Data Availability

All data relevant to the study are included in the article or uploaded as supplementary information.

## References

[1] Grobler L, Marais BJ, Mabunda S (2015). Interventions for increasing the proportion of health professionals practising in rural and other underserved areas. Cochrane Database Syst Rev.

[2] Mabunda S, Angell B, Joshi R (2021). Evaluation of the alignment of policies and practices for state-sponsored educational initiatives for sustainable health workforce solutions in selected Southern African countries: a protocol, multimethods study. BMJ Open.

[3] Mabunda SA, Angell B, Yakubu K (2020). Reformulation and strengthening of return-of-service (ROS) schemes could change the narrative on global health workforce distribution and shortages in sub-Saharan Africa. Fam Med Community Health.

[4] Mabunda SA, Durbach A, Chitha WW (2022). Are return-of-service bursaries an effective investment to build health workforce capacity? A qualitative study of key South African policymakers. PLOS Glob Public Health.

[5] Yakubu K, Durbach A, van Waes A (2022). Governance systems for skilled health worker migration, their public value and competing priorities: an interpretive Scoping review. Glob Health Action.

[6] World Health Organization (2016). Global strategy on human resources for health: workforce 2030.

[7] Kerry VB, Ahaisibwe B, Malewezi B (2022). Partnering to build human resources for health capacity in Africa: a descriptive review of the global health service partnership’s innovative model for health professional education and training from 2013-2018. Int J Health Policy Manag.

[8] World Health Organization (2019). World health statistics 2019: monitoring health for the SDGs, sustainable development goals.

[9] Asamani JA, Zurn P, Pitso P (2022). Health workforce supply, needs and financial feasibility in Lesotho: a labour market analysis. BMJ Glob Health.

[10] Dlamini CP, Khumalo T, Nkwanyana N (2020). Developing and implementing the family nurse practitioner role in Eswatini: implications for education, practice, and policy. Ann Glob Health.

[11] Roser M, Ritchie H (2019). Our world in data: HIV/AIDS. https://ourworldindata.org/hiv-aids#prevalence-in-the-total-population.

[12] (2020). Global health workforce statistics, OECD, supplemented by country data. https://data.worldbank.org/indicator/SH.MED.NUMW.P3?locations=BW-SZ-LS-NA-ZA-ZG.

[13] The World Bank (2022). Incidence of tuberculosis (per 100,000 People)-Lesotho, world. https://data.worldbank.org/indicator/SH.TBS.INCD?locations=LS-1W.

[14] Willcox ML, Peersman W, Daou P (2015). Human resources for primary health care in sub-Saharan Africa: progress or stagnation?. Hum Resour Health.

[15] Boniol M, Kunjumen T, Nair TS (2022). The global health workforce stock and distribution in 2020 and 2030: a threat to equity and ‘universal’ health coverage. BMJ Glob Health.

[16] Sachs JD, Lafortune G, Fuller G (2023). Sustainable development report 2023: implementing the SDG stimulus. https://s3.amazonaws.com/sustainabledevelopment.report/2023/sustainable-development-report-2023.pdf.

[17] Bresick G, Christians F, Makwero M (2019). Primary health care performance: a scoping review of the current state of measurement in Africa. BMJ Glob Health.

[18] Government of Botswana (1996). General orders covering the conditions of service of the public service of the Republic of Botswana.

[19] (2010). Public Service Act (act No.30 of 2008 - cap. 26:01): S.I. 19, 2010. https://uclgafrica-alga.org/wp-content/uploads/2019/05/Public-service-act-Botswana.pdf.

[20] Government of Lesotho (1978). National manpower development Council act (act No.8 of 1978).

[21] Government of Lesotho (1978). Loan Bursary fund regulations: supplement No.1 to Gazette No.29 of 11th August 1978 (legal notice No.20 of 1978).

[22] Government of Lesotho (2012). National strategic development plan 2012/13-2016/17: growth and development strategic framework.

[23] Government of the Kingdom of Eswatini (2022). The kingdom of Eswatini strategic road map: 2019-2022. https://www.cabri-sbo.org/uploads/bia/Swaziland_2019_Planning_External_NationalPlan_NatGov_COMESASADC_English.pdf.

[24] Government of the Kingdom of Swaziland (2007). The Swaziland poverty reduction strategy and action plan (PRSAP). https://www.tralac.org/files/2012/12/Final-Poverty-Reduction-Strategy-and-Action-Plan-for-Swaziland.pdf.

[25] Khumalo NS (2015). An evaluation of the “In-service Training Policy” in Swaziland with specific reference to the Ministry of Commerce.

[26] Kingdom of Lesotho Ministry of Health and Social Welfare (2004). Human resources development & strategic plan: 2005-2025. https://www.socialserviceworkforce.org/system/files/resource/files/Kingdom%20of%20Lesotho%20HR%20Development%20and%20Strategic%20Plan.pdf.

[27] Kingdom of Swaziland Ministry of Health (2012). Human resources for health strategic plan: 2012-2017. https://extranet.who.int/countryplanningcycles/sites/default/files/planning_cycle_repository/swaziland/human_resources_for_health_strategic_plan.pdf.

[28] Ministry of Health of the Kingdom of Swaziland (2012). Policy for human resources for health. https://extranet.who.int/countryplanningcycles/sites/default/files/planning_cycle_repository/swaziland/policy_for_human_resources_for_health.pdf.

[29] Mthethwa KF (2003). Training and localisation policy: a case study of Swaziland: University of the Western Cape.

[30] Nkomazana O, Peersman W, Willcox M (2014). Human resources for health in Botswana: the results of in-country database and reports analysis. Afr J Prim Health Care Fam Med.

[31] Swaziland (1965). The immigration act 1964. https://www.ilo.org/dyn/natlex/docs/ELECTRONIC/86516/97729/F480334340/SWZ86516.pdf.

[32] Swaziland Ministry of Education and Training (2011). The Swaziland education and training sector policy. https://planipolis.iiep.unesco.org/sites/default/files/ressources/swazilandeducationsectorpolicy2011.pdf.

[33] Mabunda SA, Durbach A, Chitha WW (2023). How were return-of-service schemes developed and implemented in Botswana, Eswatini and Lesotho?. Healthcare (Basel).

[34] Republic of Botswana Ministry of Health (2006). Botswana human resources strategic plan: 2007-2016.

[35] Bärnighausen T, Bloom DE (2009). Financial incentives for return of service in underserved areas: a systematic review. BMC Health Serv Res.

[36] Bärnighausen T, Bloom DE (2009). "Conditional scholarships" for HIV/AIDS health workers: educating and retaining the workforce to provide antiretroviral treatment in sub-Saharan Africa. Soc Sci Med.

[37] Bärnighausen T, Bloom DE (2009). Designing financial-incentive programmes for return of medical service in underserved areas: seven management functions. Hum Resour Health.

[38] Goma FM, Tomblin Murphy G, MacKenzie A (2014). Evaluation of recruitment and retention strategies for health workers in rural Zambia. Hum Resour Health.

[39] Gorman D (2015). Developing health care workforces for uncertain futures. Acad Med.

[40] Ncayiyana DJ (2003). The Malawi medical school--a success story. S Afr Med J.

[41] Quintana F, Sarasa NL, Cañizares O (2012). Assessment of a complementary curricular strategy for training South African physicians in a Cuban medical University. MEDICC Rev.

[42] Sui X, Reddy P, Nyembezi A (2019). Cuban medical training for South African students: a mixed methods study. BMC Med Educ.

[43] De Silva AP, Liyanage IK, De Silva STG (2013). Migration of Sri Lankan medical specialists. Hum Resour Health.

[44] Capeding TPJZ, Zarsuelo M-AM, Mendoza MAF (2020). Return service agreement in the context of the universal health care act: using International and local experiences to guide application of the RSA. Acta Med Philipp.

[45] Country watch (2010). Government structure. Lesotho Country Review.

[46] CountryWatch (2020). Botswana country review. Country report.

[47] Motsamai D (2011). Swaziland: can Southern Africa’s last absolute monarchy democratise?. African Security Review.

[48] Tong A, Sainsbury P, Craig J (2007). Consolidated criteria for reporting qualitative research (COREQ): a 32-item checklist for interviews and focus groups. Int J Qual Health Care.

[49] Asamani JA, Akogun OB, Nyoni J (2019). Towards a regional strategy for resolving the human resources for health challenges in Africa. BMJ Glob Health.

[50] Seitio-Kgokgwe O, Gauld RD, Hill PC (2016). Analysing the stewardship function in Botswana's health system: reflecting on the past, looking to the future. Int J Health Policy Manag.

[51] Cheng MH (2009). The Philippines' health worker exodus. Lancet.

[52] Pastor-Bravo M, Nelson S (2019). Migration of Latin American nurses to Spain 2006-2016: a case study. Int Nurs Rev.

[53] Lofters AK (2012). The "brain drain" of health care workers: causes, solutions and the example of Jamaica. Can J Public Health.

[54] Kupfer L, Hofman K, Jarawan R (2004). Roundtable. strategies to discourage brain drain. Bull World Health Organ.

[55] Kanchanachitra C, Lindelow M, Johnston T (2011). Human resources for health in Southeast Asia: shortages, distributional challenges, and international trade in health services. Lancet.

[56] Iredale R (1999). The need to import skilled personnel: factors favouring and hindering its international mobility. Int Migr.

[57] Asante AD, Negin J, Hall J (2012). Analysis of policy implications and challenges of the Cuban health assistance program related to human resources for health in the Pacific. Hum Resour Health.

[58] Andaya E (2009). The gift of health: socialist medical practice and shifting material and moral economies in post-Soviet Cuba. Med Anthropol Q.

[59] Huish R (2008). Going where no doctor has gone before: the role of Cuba's Latin American school of medicine in meeting the needs of some of the world's most vulnerable populations. Public Health.

[60] Alonso-Garbayo A, Maben J (2009). Internationally recruited nurses from India and the Philippines in the United kingdom: the decision to Emigrate. Hum Resour Health.

[61] Nanda PK (2021). India looks to export talent to 12 countries.

[62] Nair M, Webster P (2013). Health professionals' migration in emerging market economies: patterns, causes and possible solutions. J Public Health (Oxf).

[63] Yang C-H, Huang Y-T, Hsueh Y-SA (2013). Redistributive effects of the national health insurance on physicians in Taiwan: a natural experiment time series study. Int J Equity Health.

[64] Lehmann U, Dieleman M, Martineau T (2008). Staffing remote rural areas in middle- and low-income countries: a literature review of attraction and retention. BMC Health Serv Res.

[65] Kober K, Van Damme W (2006). Public sector nurses in Swaziland: can the downturn be reversed?. Hum Resour Health.

[66] Gow J, George G, Mwamba S (2013). An evaluation of the effectiveness of the Zambian health worker retention scheme (ZHWRS) for rural areas. Afr Health Sci.

[67] Mabunda SA, Gupta M, Chitha WW (2021). Lessons learnt during the implementation of WISN for comprehensive primary health care in India, South Africa and Peru. Int J Environ Res Public Health.

[68] Australian Government Department of Health (2013). Review of Australian government health workforce programs: achieving workforce distribution aims through return of service obligations Canberra, Australia. https://www1.health.gov.au/internet/publications/publishing.nsf/Content/work-review-australian-government-health-workforce-programs-toc~chapter-6-managing-supply-health-workers-meet-community-needs~chapter-6-achieving-workforce-distribution-aims-through-return-service-obligations.

[69] Mathews M, Heath SL, Neufeld SM (2013). Evaluation of physician return-for-service agreements in Newfoundland and Labrador. Healthc Policy.

[70] Neufeld S-M, Mathews M (2012). Canadian return-for-service Bursary programs for medical trainees. Healthc Policy.

[71] Sempowski IP (2004). Effectiveness of financial incentives in exchange for rural and Underserviced area return-of-service commitments: systematic review of the literature. Can J Rural Med.

[72] Ministry of Health and Family Welfare Government of India (2020). Agreement bond form for candidates admitted for MBBS course for 2020-2021 academic year new Delhi, India. https://mcc.nic.in/UGCounselling/Home/ShowPdf?Type=AE4F281DF5A5D0FF3CAD6371F76D5C29B6D953EC&ID=6D363479C97439B921AD2BCBA054992D8EDA9A0C&b=b&boardid=1003.

[73] Donda BM, Hift RJ, Singaram VS (2016). Assimilating South African medical students trained in Cuba into the South African medical education system: reflections from an identity perspective. BMC Med Educ.

[74] Ross AJ (2007). Success of a scholarship scheme for rural students. S Afr Med J.

