# Supplementary material for: Identifying the strengths, weaknesses, opportunities and threats (SWOT) of return-of-service bursary schemes for health workforce capacity: a qualitative study of Botswana, Lesotho and Eswatini
Source: BMJ Public Health. 2023 Oct 12;1(1):e000142. doi: 10.1136/bmjph-2023-000142 (PMC11812702; doi:10.1136/bmjph-2023-000142)
Supplement: online supplemental file 3 [file bmjph-1-1-s003.pdf]

Data collection

Four individual and two group interviews were held by via Microsoft Teams virtual platform with policymakers/implementers who were in their workplaces. Interviews took an average of 64 minutes. The design of the interview guide (Appendix 2) is described in a previous publication[2, 4]. Briefly, interviews were all conducted by the first author, and questions sought to understand the design and implementation of RoS schemes in the respective countries. The interview-guide was designed using the researchers' knowledge, literature themes and added areas of exploration. Participants gave consent for audio recording of the interviews. Transcripts were generated from Microsoft Teams and cleaned up by the first author. The first author also translated non-English language phrases (Setswana, Siswati and Sesotho) into English and interpreted acronyms used. This ensured that the final transcripts were verbatim. Member checking was undertaken by confirming the correctness of the transcript with at least one participant from each group.

Data analysis

Data were exported to NVIVO version 12 for manual analysis. A hybrid approach to inductive and deductive thematic analysis was used for analysis. First, for the inductive analysis themes were derived using a six-step approach of familiarisation, coding, theme development, review of themes, defining themes and reporting. Second, some of the codes were fitted into pre-existing themes that were derived from a similar South African analysis to finalise the theme development[4]. Third, all themes were aligned with the SWOT analysis framework which analyses the internal (strengths and weaknesses) and the external environment (opportunities and threats) in which the RoS are operating. The results are therefore presented using the SWOT analysis, 25 primary sub-themes and 7 secondary subthemes.

Reflexivity

The first author is a South African male, medical practitioner and public health medicine specialist, and a doctoral candidate in Australia during the study. He is a former RoS beneficiary from South Africa, has family in Eswatini and Botswana and has friends in all the three countries mostly from his undergraduate University years, who benefited from the three governments' RoS schemes. All the co-authors have experience in health systems and health workforce research in LMICs.

Patient and public involvement

Patients and/or the public were not involved in the design or conduct or reporting and will not be involved in the dissemination of this study as this policy is mainly relevant for health workers and policymakers.

Ethics approval

The study conformed to the principles embodied in the Declaration of Helsinki. Participants gave voluntary written consent before participating in the study. No compensation was given to participants for participating in this study. Ethics approvals were obtained from the University of New South Wales (HC200519), the Botswana Health Research and Development Division (HPDME 13/18/1), the Botswana Ministry of Tertiary Education, Research, Science and Technology (DRST 7/2/13 XXVII (27)), the Eswatini Health and Human Research Review Board (FWA 00026661/IRB 000 11253), and the Lesotho Ministry of Health Review Board (ID 24-2021). Participants have been de-identified. Direct quotes are labelled using random labels known only by the 1st author (SAM) and senior authors (BA and RJ).
